# COVID-19 related sleep disorders and the mental health

**DOI:** 10.1192/j.eurpsy.2022.1231

**Published:** 2022-09-01

**Authors:** S. Kryzhanovskiy, M. Samushia, T. Berishvili, S. Chorbinskaya, V. Shmirev

**Affiliations:** 1 Central State Medical Academy of Department of Presidential Affairs Moscow, Russia, Scientific Department, Moscow, Russian Federation; 2 Central State Medical Academy of Department of Presidential Affairs Moscow, Russia, Scientific Department, moscow, Russian Federation

**Keywords:** Disorders, sleep, Covid-19, Depression

## Abstract

**Introduction:**

COVID-19 affects nervous system and the mental health of patients.

**Objectives:**

The aim of the study was to determine the prevalence of depression, anxiety, and insomnia among patients hospitalized with COVID-19 in order to understand mediating factors and inform tailored intervention.

**Methods:**

To the study patients with mild and moderate COVID -19 were included. It was no included patients with diagnosed psychiatric disorders. It was conducted an interview, including using telemedicine technologies, assessed HADS, MFI-20, Pittsburgh Sleep Quality Index (PSQI) questionnaire.

**Results:**

It was analyzed the data of 119 patients, 34% patients was female, mean age 58,7±11,1 range 47 to 69 years. Anxiety-depressive symptoms were observed in 33/119 (28%) patients by HADS scale. Clinically significant anxiety and depression were seen in 11% and 4% of the patients, respectively. In 13% patients was observed as anxiety as depression. An increase in the MFI-20 scale (more than 20 points) was found in 87 (73.0%) patients, sleep disorders in accordance with the PSQI questionnaire was recorded in 32 (27.0%) patients. Sleep disorders were manifested by dissatisfaction with sleep quantity or quality that is associated with difficulty falling asleep. All patients have asthenic symptom.

**Conclusions:**

It was noted that in most patients with COVID-19, along with a depressive and anxiety disorders, an asthenic symptom complex, sleep disturbances are recorded. The choice of the medical intervention should be based on the severity of the violations identified taking into account the side effects of the prescribed drugs, drug interactions and somatic status of patients

**Disclosure:**

No significant relationships.

